# Sensitivity of Edge Detection Methods for Quantifying Cell Migration Assays

**DOI:** 10.1371/journal.pone.0067389

**Published:** 2013-06-24

**Authors:** Katrina K. Treloar, Matthew J. Simpson

**Affiliations:** 1 School of Mathematical Sciences, Queensland University of Technology, Brisbane, Queensland, Australia; 2 Institute of Health and Biomedical Innovation (IHBI), Queensland University of Technology, Brisbane, Queensland, Australia; University of Cambridge, United Kingdom

## Abstract

Quantitative imaging methods to analyze cell migration assays are not standardized. Here we present a suite of two-dimensional barrier assays describing the collective spreading of an initially-confined population of 3T3 fibroblast cells. To quantify the motility rate we apply two different automatic image detection methods to locate the position of the leading edge of the spreading population after 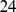, 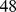 and 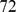 hours. These results are compared with a manual edge detection method where we systematically vary the detection threshold. Our results indicate that the observed spreading rates are very sensitive to the choice of image analysis tools and we show that a standard measure of cell migration can vary by as much as 25% for the same experimental images depending on the details of the image analysis tools. Our results imply that it is very difficult, if not impossible, to meaningfully compare previously published measures of cell migration since previous results have been obtained using different image analysis techniques and the details of these techniques are not always reported. Using a mathematical model, we provide a physical interpretation of our edge detection results. The physical interpretation is important since edge detection algorithms alone do not specify any physical measure, or physical definition, of the leading edge of the spreading population. Our modeling indicates that variations in the image threshold parameter correspond to a consistent variation in the local cell density. This means that varying the threshold parameter is equivalent to varying the location of the leading edge in the range of approximately 1–5% of the maximum cell density.

## Introduction

Cell migration plays a key role in development [1], [2], repair [3]–[5] and disease [6], [7]. Abnormalities in cell migration are associated with malignant spreading [7]–[9] and slowed wound repair [10]. Potential therapies aimed at treating these abnormalities may seek to manipulate the rate of migration by applying pharmaceutical drugs or topical treatments [8], [10], [11]. Development and validation of such therapies can be assessed by comparing assays performed under control conditions with an equivalent assay where the treatment has been applied [12]. *In vitro* migration assays can also be used to quantify the role of experimental variations such as the influence of different substrates [3], [4]. Regardless of the purpose for performing an *in vitro* cell migration assay, image detection methods that can be used to quantify the rate of cell migration are an essential element of interpreting and quantifying such assays.

Various types of assays have been used to study cell migration including two-dimensional scratch assays [3], [4] and three-dimensional Transwell assays [13], [14]. More recently, two-dimensional circular barrier assays have become a popular alternative to scratch assays [15] since they do not damage the cell monolayer, or the substrate, and are therefore thought to be more reproducible than scratch assays [8], [16]. Barrier assays are performed by placing a population of cells inside a circular barrier. The barrier is lifted and the subsequent spreading of the population is measured [17]. An essential element of interpreting and quantifying a barrier assay is to locate the position of the leading edge of the spreading population so that the rate at which the cell population spreads across the substrate can be calculated.

A common approach to quantify the cell migration rate in a barrier assay is to report the percentage change in area [15], [16], [18]–[20]. This can be expressed as
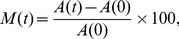
(1)where 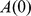 is the initial area enclosed by the population of cells, 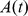 is the area enclosed by the population of cells at time 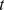, and 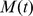 is the percentage change in area at time 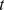.

Estimates of cell migration rates using equation (1) are often obtained by hand tracing the area enclosing the spreading cell population on an image of the assay [21], [22]. Unfortunately, hand tracing the area enclosed by the leading edge of a spreading cell population is subjective [23]. To overcome this limitation, automated image analysis software, including ImageJ [24] and MATLAB's Image Processing Toolbox [25], have become important alternatives to hand tracing [8], [26]. These software tools use edge detection and segmentation algorithms to determine the location of the leading edge of the spreading cell population. This data can then be used to quantify the cell migration rate in terms of equation (1). In addition to using automatic edge detection algorithms, it is also possible to implement user-defined edge detection options in MATLAB's Image Processing Toolbox [25] so that the user has complete control over the choice of image detection thresholds.

Since there is no standardized method for quantifying the location of the leading edge in a barrier assay, it is often difficult, if not impossible, to meaningfully compare published measures of cell migration in terms of equation (1). This difficulty is exacerbated by the fact that previously published results have been obtained using different image analysis techniques and the details are not always reported [27]–[31]. To address this limitation, here we apply three different edge detection techniques to a set of images from a two-dimensional barrier assay describing the collective spreading of a population of 3T3 fibroblast cells. We apply three different edge detection techniques to the same experimental data set and compare results from two commonly used automatic edge detection techniques and one manual edge detection technique. Our results indicate that the location of the leading edge is sensitive to the details of the edge detection procedure and this can lead to significantly different quantitative estimates of cell migration. Using a reasonable range of threshold values we show that estimates of cell migration, given by equation (1), can vary by as much as 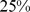 for the same data set.

To provide further insight into the edge detection techniques, we also interpret our results using a mathematical model to quantitatively describe the temporal cell spreading process associated with the barrier assay. Using previously-determined estimates of the cell diffusivity [17], we show that the location of the leading edge, as defined by the image detection methods, corresponds to contours of cell density in the range of approximately 1–5% of the maximum cell packing density. Comparing the location of the leading edge determined by the image detection methods and the mathematical model of the cell spreading provides us with a simple, but meaningful, physical interpretation of the threshold parameters used in the image detection methods.

## Materials and Methods

### 0.1 Experimental Methods

Murine fibroblast 3T3 cells (ATCC, CCL-92, Manassas, VA, USA) were grown in T175 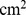 tissue culture flasks (Nunc, Thermo Scientific, Denmark) using Dulbecco's modified Eagle medium (Invitrogen, Australia) supplemented with 5% fetal calf serum (FCS) (Hyclone, New Zealand), 2mM L-glutamine (Invitrogen) and 1% v/v Penicillin/Streptomycin (Invitrogen) in 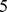
% 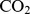 at 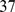
°C. Prior to confluence, cells were lifted using 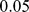 % trypsin (Invitrogen, Australia) and viable cells were counted using a Trypan blue exclusion test and a haemocytometer.

Cell migration experiments were performed using a circular barrier assay. Metal-silicone barriers, 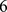 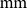 in diameter (Aix Scientifics, Germany), were cleaned, sterilized, dried and placed in the center of the wells in a 24-well tissue culture plate with 500 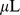 of culture medium. The wells in tissue culture plate have a diameter of 15.6 mm.

Two different densities of cell suspensions were used: 10,000 and 30,000 cells/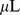. Ten 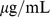 Mitomycin-C (Sigma Aldrich, Australia) was added to the cell solutions for one hour to inhibit cell proliferation [32]. One 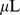 of cell suspension was carefully inserted in the barrier to ensure that the cells were approximately evenly distributed. Once seeded, the tissue culture plate was left for one hour in a humidified incubator at 37°C and 5% 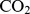 to allow the cells to attach to the surface. After the cells attached to the surface, the barriers were removed and the cell layer was washed with serum free medium (SFM; culture medium without FCS) and replaced with 0.5 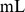 of culture medium. Plates were incubated at 37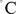 in 5% 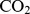 for four different times, 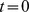, 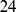, 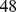 and 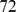 hours. Each barrier assay, for each time point, was repeated three times.

Images of the spreading cell population were obtained by fixing cells with 10% formalin, followed by 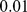
% crystal violet (Sigma-Aldrich, Australia). The stain was rinsed with phosphate-buffered saline (Invitrogen, Australia) and the plates were air-dried. Images were acquired using a stereo microscope with a Nixon digital camera (DXM1200C).

### 0.2 Edge Detection Methods

Three methods were used to detect the location of the leading edge: (i) a manual detection method written using MATLAB's Image Processing Toolbox (version 7.12) [25], (ii) an automated method using MATLAB's Image Processing Toolbox (version 7.12) [25] and (iii) an automated method using ImageJ (version 1.46r) [24]. All three methods are based on a Sobel edge detection algorithm [33] but differ in the way that the thresholds are chosen. Although different edge detection methods are available, such as the active contour method [34] and the Canny method [35], [36], we choose to focus on MATLAB and ImageJ implementations of the Sobel method since these software tools are widely available.

#### 0.2.1 Manual edge detection using the MATLAB image processing toolbox

Customized image processing software was written using the MATLAB Image Processing Toolbox [25]. The following procedure was used to detect the location of the leading edge of the spreading population. The image was imported (*imread*) and converted from color to grayscale (*rgbtogray*). The Sobel method was applied to the grayscale image by specifying a sensitivity threshold value 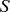, in which all edges weaker than 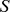 are excluded (*edge[grayscale image, ‘Sobel’,* 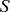]). The lines in the resulting image were dilated to show the outlines of detected edges (*strel(7), imdilate*). Remaining empty spaces in the images were filled and all objects disconnected from the leading edge were removed (*imfill, imclearborder*). The image was smoothed and filtered to remove any noise (*imerode, medfilt2*) and the area enclosed by the detected leading edge was estimated (*regionprops*).

Before we analyzed the experimental images, we undertook a preliminary step where we applied a wide range of threshold values to our experimental images, 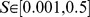. We found that thresholds in the range 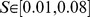 produced visually reasonable results.

#### 0.2.2 Automatic edge detection using the MATLAB Image Processing Toolbox

The manual edge detection method described in section 0.2.1 can be implemented in an automated mode by allowing the MATLAB Image Processing toolbox to automatically determine the threshold, 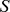, for each individual image [25]. The following procedure was used to detect the location of the leading edge. The image was imported (*imread*) and converted from color to grayscale (*rgbtogray*). The Sobel method was applied in the automatic mode (*edge[grayscale image, ‘Sobel’*]). The lines in the resulting image were dilated (*strel(7), imdilate*). Remaining empty spaces were filled and all objects disconnected from the leading edge were removed (*imfill, imclearborder*). The image was smoothed and filtered (*imerode, medfilt2*) and the area enclosed by the detected leading edge was estimated (*regionprops*).

#### 0.2.3 Automatic edge detection using ImageJ

ImageJ software [24] was used to automatically detect the position of the leading edge. For all images, the image scale was set (*Analyze*-*Set scale*) and color images were converted to grayscale (*Image*-*Type*-*32bit*). The Sobel method was used to enhance edges (*Process*-*Find Edges*). The image was sharpened (*Process*-*Find Edges*) and an automatically determined threshold was applied (*Image*-*Adjust*-*Threshold*-*B&W*-*Apply*). After applying the Sobel method again (*Process*-*Find Edges*), the wand tracing tool, located in the main icons box, was used to select the detected leading edge. The area enclosed by the detected leading edge was calculated (*Analyze*-*Set Measurements*-*area, Analyze*-*Measure*).

### 0.3 Mathematical Modeling Tools

To provide a physical interpretation of our image analysis results, we use a mathematical model to relate the edge detection results to the spatial distribution of the cell density. We model the spreading population of cells using a linear diffusion equation [3]–[5], with previously determined values of the cell diffusivity [17]. The effects of cell proliferation are neglected in our mathematical model, and this is consistent with our experimental protocol where cells were pretreated with Mitomycin-C to suppress cell proliferation [32].

To relate our edge detection results to the cell density, we consider the solution of the two-dimensional axisymmetric diffusion equation.
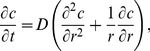
(2)where 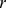 is radial position, 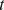 is time, 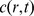 is the non-dimensional cell density and 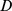 is the cell diffusivity, which is a measure of random, undirected, cell motility [17], [37]. The non-dimensional cell density is obtained by scaling the dimensional cell density, 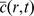, by the carrying capacity density 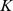. This gives 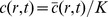, with 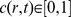. The carrying capacity density is estimated by assuming that the maximum packing density of cells corresponds to a square packing density. The average cell diameter is 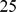 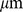, giving 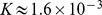 cells per 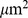 [17].

We solve equation (2) on the domain 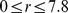 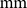. The boundary at 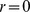 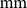 corresponds to the center of the well and we apply a symmetry condition, 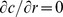, here [38]. The boundary at 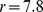 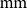 corresponds to the outer edge of the well which is a physical boundary and so we apply a zero flux boundary condition here. The boundary condition at 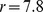 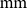 is irrelevant for our barrier assay results since the leading edge of the spreading cell front did not reach this boundary on the time scale of the experiments [17]. The initial condition is given by,
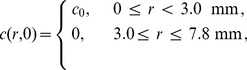
(3)where 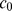 is the density of cells initially inside the barrier. Assuming that the cells have an average diameter of 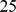 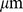 [17], we can pack 3000/25 cells across the radius of the barrier. Hence, we estimate that the maximum number of cells that can be packed in a monolayer in the barrier is 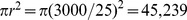. To specify the initial condition using for equation (3), we assume that either 10,000 or 30,000 cells are uniformly distributed within the barrier giving 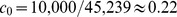 and 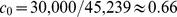, respectively.

Numerical solutions of equation (2) are obtained using a finite-difference approximation on a grid with a uniform grid spacing of width 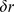, and implicit Euler stepping with uniform time steps of duration 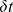 [39], [40].

## Results

### 0.4 Locating the Leading Edge

**Figure 1:**
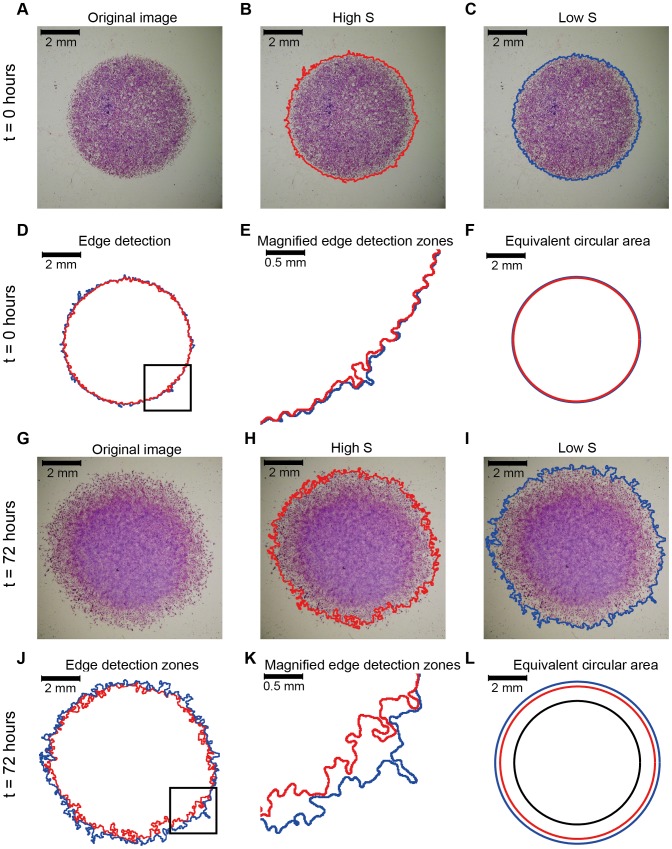
Locating the leading edge in a barrier assay. Images of barrier assays containing 30,000 cells at 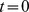 hours (A–F) and 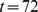 hours (G–L). (A,G): Images from the barrier assay. (B,H): Leading edge for a high threshold 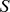 in red, superimposed on an image of the spreading population. (C,I): Leading edge for a low threshold 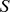 in blue, superimposed on the an image of the spreading population. (D,J): Comparing high and low 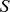 detected edges at 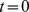 hours. (E,K): Detailed comparison of the detected edges in the boxed area in D and J. (F,L): Comparing equivalent circular areas. The black line in (L) shows the initial circular area. Scales are given in each subfigure.

To demonstrate the sensitivity of different image processing tools, we apply the manual edge detection method, with different threshold values, to images showing the entire spreading populations in several different barrier assays. Images in Fig. 1A and Fig. 1G show the spreading population in a barrier assay with 30,000 cells at 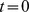 and 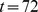 hours, respectively. Visually, the leading edge of the cell population at 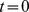 (Fig. 1A) appears to be relatively sharp and well-defined. In contrast, the leading edge of the cell population at 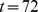 hours (Fig. 1G) is diffuse and less well-defined. This indicates that is it difficult to visually identify the location of the leading edge after the barrier has been lifted and the cell population spreads outwards, away from the initially-confined location.

Our visual interpretation of the images indicate that the precise location of the leading edge is not always straightforward to define. To explore this subjectivity, we use the manual edge detection method (section 0.2.1) by specifying different values of the Sobel threshold, 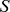. Results in Fig. 1B and Fig. 1C show the detected leading edges at 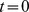 hours using a high threshold (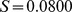) and a low threshold (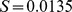), respectively. For both thresholds, the detected leading edges appear to be appropriate representations of the leading edge of the spreading population, and are very similar to each other. Results in Fig. 1H and Fig. 1I show the detected leading edges at 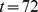 hours for a high threshold (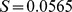) and a low threshold (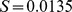), respectively. Both detected edges at 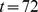 hours appear to be reasonable approximations to the location of the leading edge of the spreading population, however they are very different to each other which indicates that the results are sensitive to 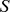.

To qualitatively compare the two leading edges detected at 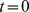 hours (Fig. 1B and Fig. 1C) we superimpose the two detected leading edges in Fig. 1D and show a magnified portion of these edges in Fig. 1E. The superimposed edges confirm that the choice of 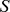 has relatively little influence at 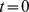 hours. We now compare equivalent results at 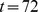 hours from Fig. 1H and Fig. 1I. Superimposing the two leading edges for high and low 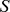 thresholds in Fig. 1J indicates that there is a distinct difference between them. A magnified portion of the detected leading edges is shown in Fig. 1K which also supports our initial observation that it is difficult to visually delineate the leading edge of the spreading population when the leading edge is diffuse.

Our edge detection results at 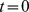 hours and 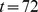 hours, in Fig. 1A–E and Fig. 1G–K, qualitatively indicate that the threshold value is important in detecting the edge at a later time. To quantitatively compare our edge detection results, we calculate the area enclosed by the detected leading edge and convert this area into an equivalent circle with radius 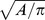. Results in Fig. 1F show the equivalent circular areas for low and high thresholds at 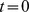 hours. The area of the low and high thresholds are 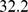 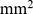 and 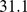 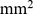, respectively, giving a relatively small difference of 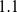 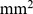. These two circles are almost visually indistinguishable at the scale shown in Fig. 1F, confirming there is very relatively little difference regardless of the threshold. Equivalent circular areas in Fig. 1L show the low and high threshold areas at 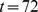 hours superimposed on the initial area. The area of the two outer circles in Fig. 1L is 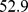 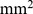 and 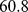 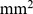, giving a relatively large difference of 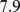 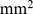. If we take the initial area to be 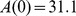 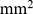 then equation (1) gives us 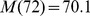
% for the high threshold leading edge in Fig. 1H and 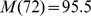
% for the low threshold leading edge in Fig. 1I. These results indicate that the increase in area enclosed within the leading edge of the spreading cell population is very sensitive to the choice of threshold and the results can vary by as much as 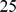
%.

### 0.5 Comparing Edge Detection Techniques

To explore and quantify the sensitivity in detecting the leading edge for our barrier assays, we now extend our initial investigation and detect the location of the leading edge across all experimental images acquired at different time points. We applied the manual edge detection technique to all images using thresholds within the range 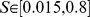. For each threshold value, we calculated the area enclosed by the detected leading edge and we analyzed the images from each experimental replicate separately so that we could calculate the mean area enclosed by the leading edge, 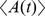. We estimated the variability amongst the experimental replicates by calculating the standard deviation about the mean, 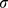. Our results are summarised in Table 1, where we see that the variability amongst the experimental replicates is small with typical values of 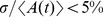. From this point onward we will report all our experimental results in terms of the mean area, 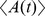, and for convenience we will drop the angle bracket notation.

**Table 1:** Edge detection area (mm^2^) results.

Number of Cells	Time (hours)	Mean Area Manual S High (mm^2^)	Standard Deviation ManualS High (mm^2^)	Mean Area Manual S Low (mm^2^)	Standard Deviation Manual S Low (mm^2^)	Mean Area Auto ImageJ (mm^2^)	Standard Deviation Auto ImageJ (mm^2^)	Mean Area Auto Matlab (mm^2^)	Standard Deviation Auto Matlab (mm^2^)
10,000	0	27.4	0.67	30.1	1.61	30.3	0.83	29.0	1.64
	24	31.9	0.91	36.0	0.63	35.0	2.26	34.2	0.78
	48	36.2	1.91	43.4	0.68	41.3	1.11	39.1	2.64
	72	39.7	1.98	47.1	0.62	45.8	0.81	44.6	0.81
30,000	0	31.1	0.21	33.5	0.34	33.1	1.40	30.0	1.56
	24	44.8	2.11	50.3	1.08	49.9	1.40	45.0	2.12
	48	50.0	1.52	55.5	1.78	55.2	1.57	51.4	1.47
	72	52.9	2.25	60.8	2.11	55.9	3.01	54.6	3.10

Summary of edge detection results comparing the manual edge detection technique (Manual), the MATLAB Image Processing Toolbox automatic technique (Auto MATLAB) and the ImageJ automatic technique (Auto ImageJ). All results show the average area estimated using three identically-prepared and analyzed experimental replicates. The variability amongst experimental replicates is estimated using the standard deviation.

**Figure 2:**
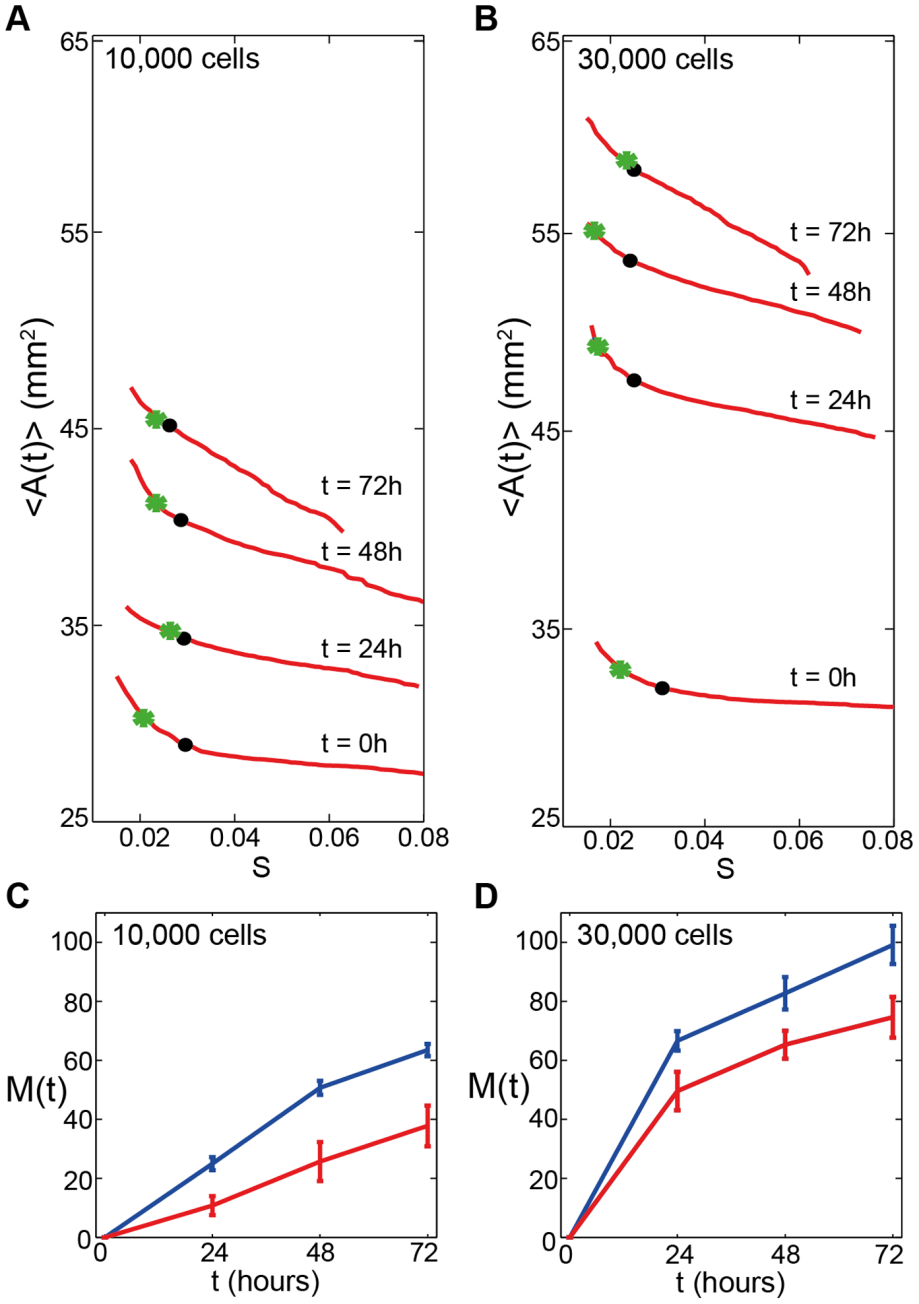
Comparing edge detection techniques. Comparing three edge detection techniques for barrier assays with two different cell densities: 10,000 cells (A,C) and 30,000 (B,D) cells. (A–B): Comparison of the three edge detection techniques showing the mean area enclosed by the leading detected edge at 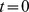, 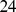, 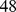 and 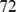 hours with time points indicated. Red lines correspond to the the manual edge technique using MATLAB's Image Processing Toolbox for a range of the threshold parameter 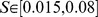. Black dots correspond to the automatic MATLAB results and the green asterisks correspond to the automatic ImageJ results. (C–D): The migration rate of cells in the barrier assays expressed as 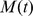
% using equation (1). Results correspond to the minimum (red) and maximum (blue) average areas detected using the manual MATLAB technique. Error bars correspond to one standard deviation about the mean.

We now compare the sensitivity of our manual edge detection results by analyzing the images at using a range of threshold values for several different time points for barrier assays with two different initial cell densities. Results in Fig. 2A and Fig. 2B show the relationship between the average area enclosed by the detected leading edge and the threshold value 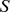 for a barrier assay with 10,000 and 30,000 cells, respectively. Initially, for the barrier assay with 10,000 cells, the minimum average area enclosed by the detected leading edge is 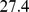 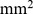 and the maximum area is 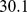 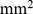. For the barrier assay with 30,000 cells, the minimum and maximum initial average area enclosed by the detected leading edge is 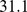 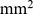 and 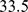 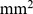, respectively. For both initial cell densities, the difference between the maximum and minimum detected initial area is relatively small compared to the differences we observe at later times, as we will now demonstrate.

Results in Fig. 2A and Fig. 2B show that the average area enclosed by the detected leading edges increases with time as the cell population spreads outwards from the barrier. We expect that the sensitivity in detecting the location of the leading edge will increase with time as the population spreads and the leading edge becomes increasingly diffuse. For the barrier assays initialized with 10,000 cells, results in Fig. 2A show that the minimum area detected at 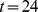 hours is 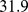 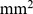 and the maximum area detected is 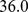 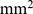, giving a difference of  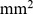. At 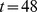 hours the minimum area is 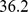 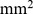 and the maximum area is 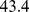 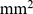, giving a difference of 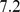 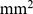. At 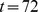 hours, the minimum area is 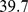 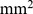 and the maximum area is 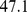 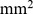, giving a relatively large difference of 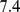 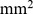. These results indicate that the sensitivity in detecting the leading edge is relatively large and that the results depend on the choice of the threshold, and this sensitivity increases with time as the leading edge of the spreading population becomes increasingly diffuse.

Equivalent manual edge detection results for barrier assays containing 30,000 cells in Fig. 2B show similar trends to the results previously reported for the barrier assays with 10,000 cells. The minimum detected average areas at 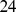, 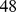 and 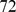 hours are 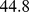 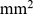, 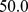 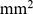 and 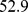 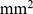, while the maximum detected average areas are 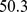 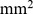, 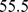 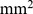 and 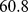 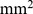, respectively. Comparing the minimum and maximum average areas for the barrier assay with 30,000 cells gives differences of 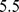 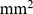, 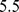 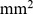 and 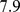 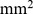 at 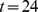, 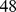 and 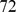 hours, respectively.

Our results using the manual edge detection method illustrate that there are many plausible approximations of the leading edge of the spreading populations for a range of threshold values. We now compare the manual edge detection algorithm with two automatic edge detection methods. We applied the automatic MATLAB and ImageJ techniques (section 0.2.3 and section 0.2.2), to the same images we previously analysed using the manual edge detection method. For both automatic techniques, the average area enclosed by the detected edge was calculated and compared to the average areas obtained using the manual edge detection method. Results in Fig. 2A and Fig. 2B show the automatic edge detection results relative to the manual results, and estimates of the mean and standard deviation of the area obtained using the automatic techniques are given in Table 1. The MATLAB and ImageJ results confirm that both automatic techniques give estimates that are consistent with those obtained using the manual edge detection method. However, the automatic techniques are restricted in the sense that they can only detect one particular location whereas the manual edge detection method can produce many different results, all of which are reasonable estimates of the position of the leading edge of the spreading cell population.

We now use equation (1) to quantify the observed cell migration in our barrier assays. This approach requires that we use an estimate of 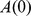, the initial average area. Our previous results indicate that the initial average area of the spreading population ranged from 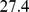 to 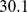 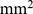 for the barrier assay with 10,000 cells while the initial average area of the spreading population ranged from 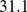 to 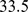 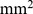. To estimate 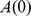 we will take the average of these maximum and minimum estimates so that we have 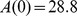 and 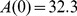 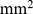 for the barrier assays with 10,000 and 30,000 cells, respectively. To estimate the sensitivity of our results as a function of the threshold value in the manual edge detection technique, we apply equation (1) using the minimum and maximum detected average areas from our manual edge detection method. The details of the results for all three edge detection techniques are given in Table 2. Although we observe that the two automatic methods produce similar results for certain assays at certain times, the differences between the results for the two automatic edge detection methods can be very large with 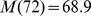 % for the barrier assay with 30,000 cells according to the ImageJ results whereas 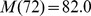 % for the same assay according to the automatic MATLAB method. Profiles in Fig. 2C and Fig. 2D show how 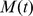 varies with time according to the results obtained from the manual edge detection method applied to the images from the barrier assays initialized with 10,000 and 30,000 cells, respectively. Figure 2C and Fig. 2D each contain two sets of results corresponding to the average estimate of 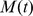 calculated using the low 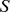 threshold, and the average estimate of 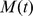 calculated using the high 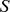 threshold. The differences between the low and high threshold results in Fig. 2C is 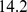 %, 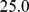 % and 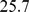 % for 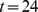, 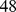 and 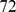 hours, respectively. The difference between the low and high threshold results in Fig. 2D (30,000 cells) is 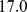 %, 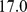 % and 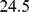 % for 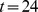, 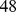 and 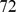 hours, respectively. These results indicate that estimates of cell migration using equation (1) are very sensitive to the details of the edge detection technique and that this sensitivity increases with time.

**Table 2:** Quantifying the cell migration rate using equation (1).

Number of Cells	Time (hours)	M(t) Manual S High	M(t) Manual S Low	M(t) Auto ImageJ	M(t) Auto Matlab
10, 000	24	10.8	25.0	14.4	17.9
	48	25.7	50.7	35.0	34.8
	72	37.8	63.5	49.7	53.8
30,000	24	49.6	66.6	50.8	50.0
	48	65.6	82.7	66.8	71.3
	72	74.6	99.1	68.9	82.0

The cell migration rate in terms of M(t) using equation (1) and the average area results from Table 1. Results are reported for the manual edge detection technique with a high threshold (Manual S high), the manual edge detection technique with a low threshold (Manual S Low), the MATLAB Image Processing Toolbox automatic technique (Auto MATLAB) and the ImageJ automatic technique (Auto ImageJ).

### 0.6 A Physical Interpretation of the Leading Edge

**Figure 3:**
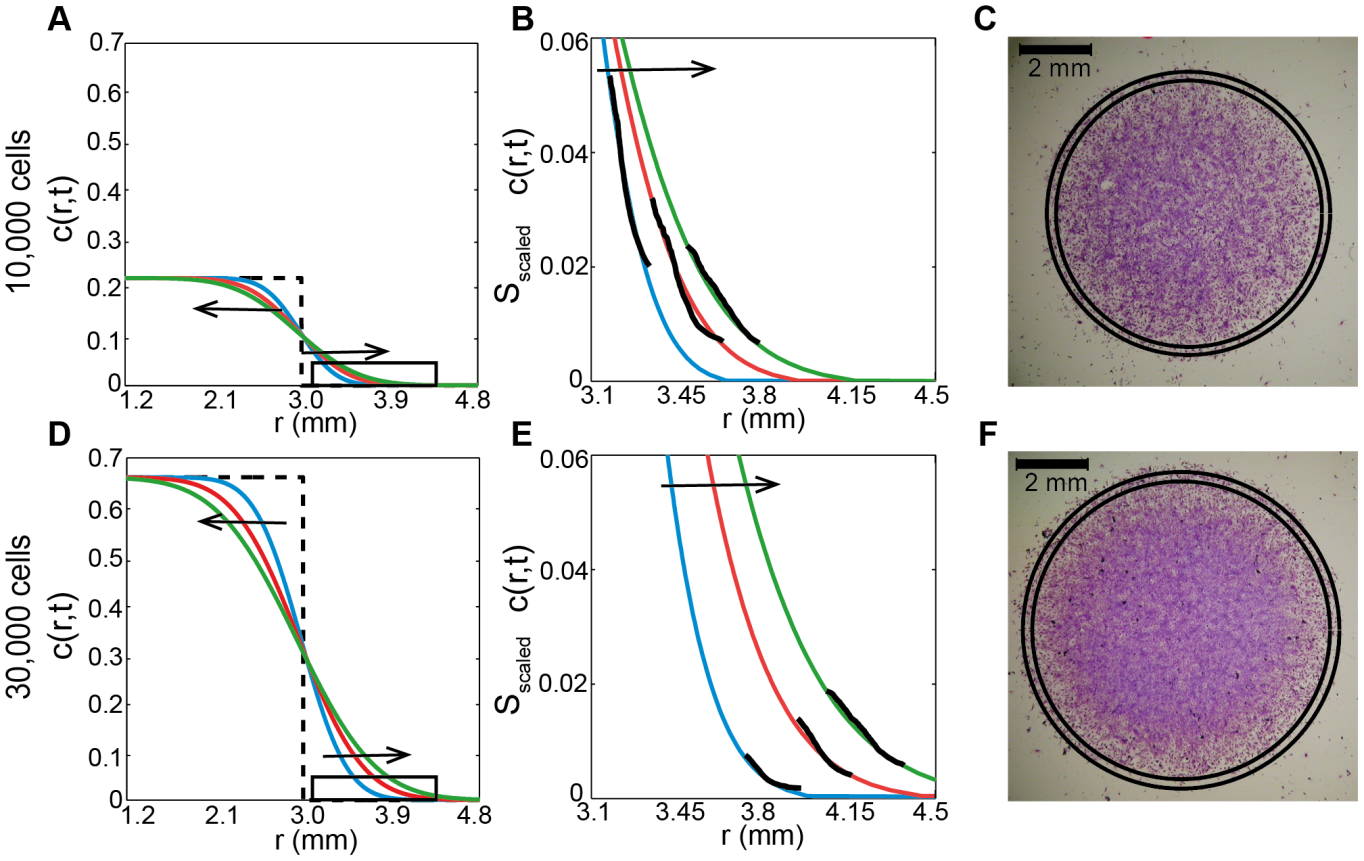
Physical interpretation of the edge detection results. (A, D): Solutions of equation (2) showing the density profiles near the leading edge at 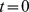 (dotted black), 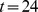 (blue), 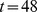 (red) and 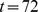 hours (green). Arrows indicate the direction of increasing time. The initial conditions is given by equation (3) with 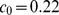 and 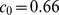 for barrier assays with 10,000 and 30,000 cells, respectively. Numerical solutions of equation (2) are obtained with 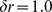 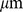 and 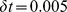 hours, with 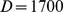 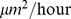 and 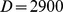 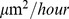 for barrier assays with 10,000 and 30,000 cells, respectively. (B,E) The detail of the solutions of equation (2) from the boxed area in (A,D) compared with the scaled manual edge detection results (black) from Figure 2 (A,C). (C,F) Images of a barrier assay with 10,000 and 30,000 cells at 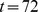 hours, respectively. The contours of the solution of equation (2) are superimposed. The values of the contours are 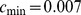 and 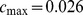 for the barrier assay with 10,000 cells, and 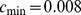 and 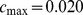 for the barrier assay with 30,000 cells.

Previously, we used three different edge detection techniques to determine the location of the leading edge of spreading cell populations in several barrier assays. Although these techniques produce visually reasonable approximations to the position of the leading edges, the techniques do not give us any physical measure, or definition, of the leading edge. To address this, we now interpret our edge detection results using a mathematical model of the cell spreading process. For each barrier assay experiment, we solve equation (2) using the appropriate boundary and initial conditions (section 0.3) and previous estimates of the cell diffusivity [17]. The solution profiles in Fig. 3A and Fig. 3D, show the predicted cell density near the leading edge of the spreading cell populations in the barrier assay at 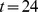, 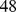 and 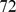 hours. The difference between the two initial cell densities in the barrier assays is shown in these profiles since we have 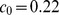 in the center of the barriers for the assays initialized with 10,000 cells (Fig. 3A) whereas we have 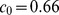 in the center of the barriers for the assays initialized with 30,000 cells (Fig. 3D).

To determine a physical relationship between the threshold value 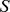 and the cell density at the corresponding detected edge, we compare our manual edge detection results to solutions of equation (2). For each set of averaged edge detection results, we scale the threshold values to match the corresponding solution of equation (2). The scaling is given by.
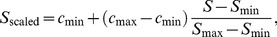
(4)where 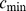 and 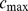 are the minimum and maximum contours of the solution of equation (2), 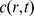, which enclose the same average area detected by the manual edge detection method applied with the minimum and maximum thresholds, 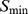 and 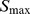, respectively.

Profiles in Fig. 3B and Fig. 3E compare the scaled edge detection results to corresponding solutions of equation (2) at 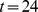, 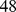 and 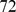 hours for barrier assays with 10,000 and 30,000 cells, respectively. For both initial density experiments at all time points, the shape of the 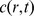 density profiles matches the shape of the edge detection results. This match indicates that varying the threshold value 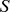 corresponds to a consistent variation in the spatial distribution of cell density in the spreading cell population. Comparing the edge detection results to the corresponding contours of the cell density, we observe that the manual edge detection technique identifies a range of leading edges corresponding to cell densities of 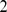
–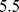 % at 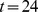 hours, 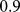
–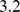 % at 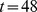 hours and 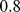
–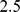 % at 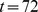 hours for the barrier assays with 10,000 cells. Equivalent results in Fig. 3E indicates that the manual edge detection technique identifies a range of leading edges corresponding to cell densities of 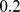
–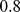 %, 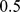
–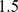 % and 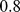
–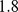 %, for 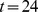, 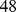, 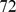 hours for the barrier assay with 30,000 cells. In summary, the manual edge detection technique identifies a range of leading edges corresponding to cell densities of approximately 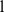
–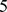 % of the maximum packing density.

The images in Fig. 3C and Fig. 3F show snapshots from two barrier assays at 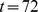 hours with 10,000 and 30,000 cells, respectively. To illustrate the location of the leading edge, defined by contoured solutions of equation (2), we superimpose the 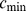 and 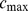 contour of the appropriate solution of equation (2). In both cases we observe that the 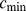 and 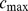 contours are reasonable approximations to the location of the position of the leading edge of the spreading populations. In each experiment, the difference between the 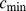 and 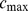 contours are relatively large and this recapitulates the sensitivity observed previously in Fig. 1H and Fig. 1I.

## Discussion and Conclusions

Cell migration is an essential aspect of development [1], [2], repair [3]–[5] and disease [6], [7]. *In vitro* cell migration assays are routinely used to assess the migration potential of different cell types [8], [9] as well as assessing the potential for different types of treatment strategies aimed at regulating cell migration [10]–[12], [16]. Currently, many studies report results from cell migration assays without specifying the details of how the assays are measured or interpreted [27]–[31]. In an attempt to address this limitation we compare three different image processing techniques to quantify the migration rate of cells in a two-dimensional barrier assay [17]. Our visual interpretation of the images from the barrier assays indicate that the position of the leading edge of the spreading population is relatively sharp and well-defined at the beginning of the assay. However, we observe that the leading edge of the spreading cell population becomes increasingly diffuse and less well-defined at later times as the cell population spreads across the substrate. We quantify the rate of cell migration using a standard measure, given by equation (1), describing how the area enclosed by the leading edge of the spreading population increases with time. To explore how such a standard measure of cell migration depends on the edge detection methods we calculate the location of the leading edge of the spreading population using three different image processing tools. In summary, our results indicate that estimates of the cell migration rate are very sensitive to the details of the image processing tools and we show that our estimates of the cell migration rate can vary by as much as 25% for the same data set. These differences depend on the choice of threshold used in the edge detection technique. Our measurements indicate that the concept of the area enclosed by the leading edge is poorly defined and we suggest that one way to overcome these difficulties is to use a direct measurement of cell density. For example, a nuclear stain could be used to reveal the locations of individual cells within the spreading population [17].

In addition to comparing estimates of cell migration using different image processing techniques, we also provide a physical interpretation of the results from the manual edge detection technique by using a mathematical model of the cell spreading process. We use a previously-parameterised [17] mathematical model to describe the spatial and temporal variation in cell density associated with the barrier assays and we compare our modelling results with the edge detection results. For all images processed by the manual edge detection technique, we identified a range of Sobel threshold values, from 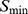 to 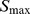, that could be used to produce a reasonable estimate of the location of the leading edge of the spreading populations. We scaled these values so that they corresponded with a range of cell density contours, from 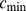 to 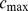, corresponding to the minimum and maximum contours of the relevant solution of equation (2). Our results indicate that varying the threshold 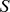 corresponds to a consistent variation in the spatial distribution of cell density in the spreading cell population. In particular, the manual edge detection technique identifies the leading edge of the population within a range of the cell density of approximately 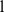-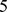
% of the maximum packing density. The close match between the position of the leading edge as a function of the Sobel threshold and the solution of the partial differential equation describing the spreading process suggests that this type of information could be used to estimate the diffusivity of the cells, 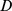. This could be a useful method for estimating the cell diffusivity since it is well known that estimates of cell diffusivity can vary by as much as an order of magnitude and these variations depend on the kind of cell and the substrate being considered [41].

As a result of this study, we recommend that the location of the leading edge of a spreading cell population in a cell migration assay should not be determined using any kind of hand tracing technique. Instead, a computational image processing technique should be used to reduce the impact of the subjectivity of the analyst. Our results demonstrate that the computational edge detection techniques can be very sensitive to the choice of threshold applied to the image. Therefore, we recommend that images of cell migration assays should be analysed using a manual edge detection technique and that the details of the image thresholds should be reported.

We anticipate that our results for the two-dimensional barrier assay will also be relevant to other types of cell migration assays such as scratch assays [3], [4], or different types of circular barrier assays that include the outward migration of cells away from an initially-confined circular population [17] as well as barrier assays describing the inward migration of cell populations into an initially-vacant circular region [8], [9], [16]. We also expect that our results for the two-dimensional barrier assay could be extended by considering other types of experimental conditions. For example, here we chose to present results for cells that were pretreated to prevent cell proliferation [32] so that we could study cell spreading processes driven by cell migration alone in the absence of cell proliferation. Given that the shape of the leading edge of the spreading cell population depends on the relative contribution of cell migration and cell proliferation [6], [17], we expect that comparing different edge detection results for different cell populations with different relative rates of cell proliferation and cell migration will also be of interest [37], [42]. Finally, although we have presented our image analysis techniques in the context of analyzing an *in vitro* cell migration assay, these concepts will also be relevant when considering *in vivo* cell spreading, such as in the detection of the leading edge of spreading melanomas [34], [43].
